# Impact of the comprehensive geriatric assessment on treatment decision in geriatric oncology

**DOI:** 10.1186/s12885-020-06878-2

**Published:** 2020-05-06

**Authors:** Sandrine Sourdet, Delphine Brechemier, Zara Steinmeyer, Stephane Gerard, Laurent Balardy

**Affiliations:** 1 Gérontopôle, Department of Internal Medicine and Geriatrics, Toulouse University Hospital, La Cité de la Santé, Hôpital La Grave, Place Lange, TSA 60033, 31059 Toulouse, Cedex 9 France; 2 UMR Inserm Unit 1027, University of Toulouse III, Toulouse, France

**Keywords:** Geriatric oncology, Geriatric assessment, Treatment decision-making, Physical performance, Cognitive impairment, Malnutrition

## Abstract

**Background:**

The comprehensive geriatric assessment (CGA) is the gold standard in geriatric oncology to identify patients at high risk of adverse outcomes and optimize cancer and overall management. Many studies have demonstrated that CGA could modify oncologic treatment decision. However, there is little knowledge on which domains of the CGA are associated with this change. Moreover, the impact of frailty and physical performance on change in cancer treatment plan has been rarely assessed.

**Methods:**

This is a cross-sectional study of older patients with solid or hematologic cancer referred by oncologists for a geriatric evaluation before cancer treatment. A comprehensive geriatric assessment was performed by a multidisciplinary team to provide guidance for treatment decision. We performed a multivariate analysis to identify CGA domains associated with change in cancer treatment plan.

**Results:**

Four hundred eighteen patients, mean age 82.8 ± 5.5, were included between October 2011 and January 2016, and 384 of them were referred with an initial cancer treatment plan. This initial cancer treatment plan was changed in 64 patients (16.7%). In multivariate analysis, CGA domains associated with change in cancer treatment plan were cognitive impairment according to the MMSE score (*p* = 0.020), malnutrition according to the MNA score (*p* = 0.023), and low physical performance according to the Short Physical Performance Battery (*p* = 0.010).

**Conclusion:**

Cognition, malnutrition and low physical performance are significantly associated with change in cancer treatment plan in older adults with cancer. More studies are needed to evaluate their association with survival, treatment toxicity and quality of life. The role of physical performance should be specifically explored.

## Background

There is rising incidence and prevalence of cancer in older adults worldwide. In 2017, France, 65% of all cancers were diagnosed in older adults aged 65 years and above [1]. By 2030, 70% of all cancers will occur in patients aged over 65 years in the USA [2]. Because of the complexity of this population, the management of older adults with cancer is challenging. Oncologic treatment is often based on chronological age [3], but it does not reflect the heterogeneity of this population and predicts poorly treatment tolerance. There is growing evidence that the decision-making process should be rather based on patient’s functional age [4].

The comprehensive geriatric assessment (CGA) is an effective tool for assessing a patient’s functional age. It has been proven to be a strong predictor of adverse events in geriatric oncology patients, and is recommended in treatment decision making by the International Society of Geriatric Oncology (SIOG) [5]. According to the SIOG, the following domains of the CGA need to be evaluated: functional status, comorbidity, medication, cognition, fatigue, psychosocial status, nutrition and geriatric syndromes assessment [5]. In addition to this usual assessment, frailty assessment is increasingly recommended in the oncogeriatric approach and reflects well functional reserve [6]. Frailty is a state of vulnerability to poor resolution of homeostasis following stress. In the oncogeriatric population, frailty is associated with poor outcomes such as surgical complications, chemotherapy and radiotherapy morbidity and mortality [6, 7]. Many tools have been developed to identify frailty: the most common being the Fried’s criteria [8], and the frailty Index [9], but physical performance tests such as gait speed, or the short physical performance Battery (SPPB) may perform as well [10]. Poor SPPB score, gait speed, or Timed Up & Go (TUG) Test are associated with mortality, treatment complications, and functional decline [11–13]. Nevertheless they have been insufficiently studied in the treatment decision process in geriatric oncology.

The CGA has been recommended in oncology practice for many reasons: identification of health problems usually not screened during routine oncological assessment, implementing non-oncologic interventions, but also change in cancer treatment plan. In a recent review, Hamaker et al. showed that the CGA modified the oncologic treatment plan in 8 to 54% of patients [14]. But there is little knowledge on which CGA domain could influence changes in cancer treatment decision. Only a few studies have tried to identify CGA parameters associated with change in cancer treatment plan [14]. Frailty and physical performance are rarely assessed.

In this study, we aimed to identify domains of CGA associated with change in cancer treatment plan in older patients with cancer, including frailty and physical performance.

## Method

In Toulouse University Hospital, a geriatric consultation team including a geriatrician experienced in oncology (with an university degree in geriatric oncology) and a geriatric nurse, can be requested by an oncologist, surgeon or radiation therapist, to provide a geriatric expertize in older patients with cancer in various hospital units [15]. They perform a one-hour geriatric assessment at the patient’s bedside and give conclusions about geriatric impairment, subsequent interventions, and if needed they can provide guidance for cancer treatment decision. In complex clinical situations or treatment plans, a more complete geriatric evaluation may be advised so the oncogeriatric patients are referred to the Geriatric Frailty Clinic.

The geriatric frailty clinic (GFC) is a geriatric day hospital of the Gerontopole of Toulouse, France, dedicated to the prevention of disability in frail older patients. It also evaluates patients aged 65 years and older with solid or hematological cancer during a pre-therapeutic evaluation. Its organization and overall activity are well described elsewhere [16]. Each patient undergoes a CGA performed by a multidisciplinary geriatric team (including a geriatrist or a general practitioner specifically trained in geriatrics, a nurse, a nurse-aid, an orthoptist (paramedical profession specialized in the eye care sector), and if needed a dietician, a neuropsychologist, and a physical activity teacher). In geriatric oncology patients, a geriatrician specialized in oncology is also consulted.

**Fig. 1:**
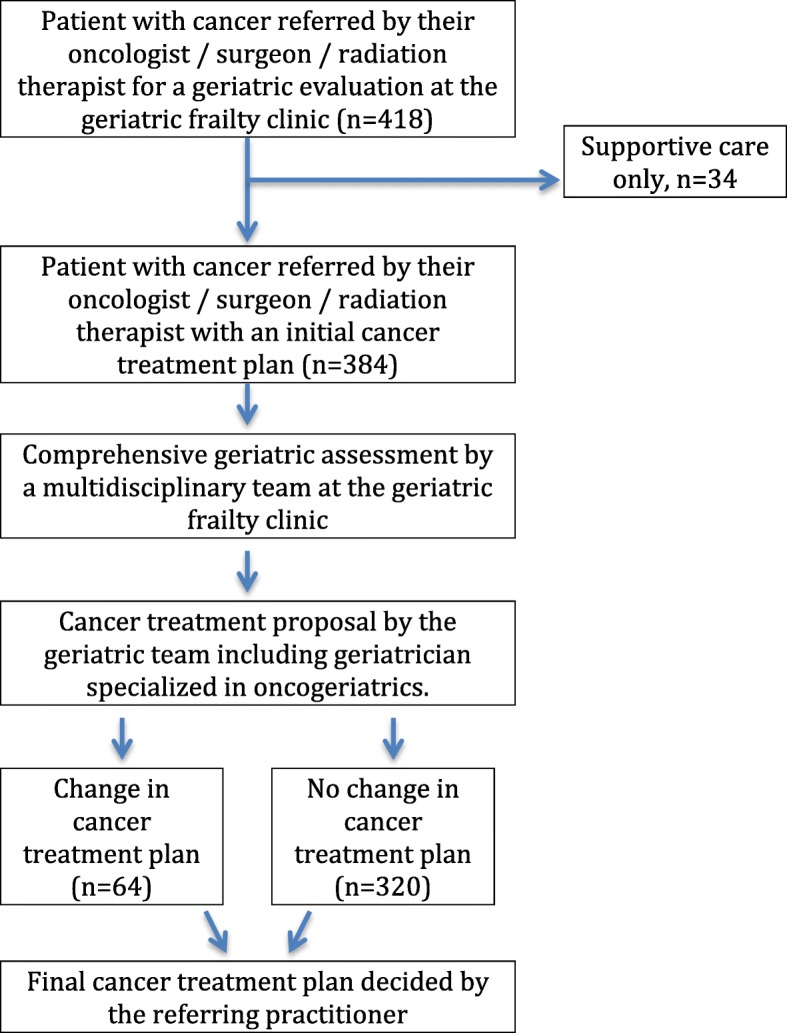
Decision making-process for the older patients with cancer referred to the geriatric frailty clinic

The first objective of the evaluation is to provide guidance concerning cancer treatment decision. The CGA is a recommended assessment able to: 1/ give helpful information concerning the existence of unidentified health-related problems and geriatric syndromes, 2/ help to estimate life-expectancy in the context of cancer, comorbidities and geriatric status, 3/ predict treatment-related complications and overall survival [5]. At the end of the evaluation, during a multi-professional meeting, the geriatric team and the geriatrician specialized in oncology, propose to maintain or change the initial cancer treatment plan, according to the conclusions of the CGA. Changes in cancer treatment may be graded as follow: intensification of cancer treatment, decrease in treatment intensity or change from specific cancer treatment to supportive care. In case of change, the geriatric proposal is discussed with the referring practitioner, who will decide the final treatment. The decision-making process is described in Fig. 1. A second objective of the CGA is to propose therapeutic and non-therapeutic interventions to optimize the patient’s health status before the cancer treatment.

In this analysis, oncogeriatric patients evaluated between October 2011 and January 2016 were included. Ethics approval for this study was obtained from the local ethic committee in Toulouse University Hospital.

### Comprehensive geriatric assessment

A CGA was performed for all consecutive patients. Social environment assessment included living conditions and marital status. A medical evaluation recorded the type and stage of cancer. Comorbidities were assessed according to the Charlson Comorbidity Index [17]. Polypharmacy was defined as five or more prescribed medications [18]. Functional abilities were assessed using Kat’z Activities of Daily Living (ADL) and Lawton’s Instrumental Activities of Daily Living [19, 20]. Frailty and physical function were measured respectively using Fried’s Criteria [8] and the Short Physical Performance Battery (SPPB) [21]. Frailty criteria included 1) unintended weight loss, 2) self-reported exhaustion, 3) low hand-grip strength (as measured by a dynamometer and stratified by BMI and sex), 4) slow walking speed (4 m usual walk speed stratified by height and sex) and 5) low physical activity. Patients were classified as frail if they met at least three criteria, pre-frail if they met one or two criteria, and robust if they met no criteria. The SPPB consists of three measurements: 1) standing balance test, 2) four meters walking speed and, 3) chair stand. Patients were categorized into three groups according to the SPPB score: high performance (score 10–12), medium performance (score 6–9), low performance (score 0–6). The G-8 geriatric screening tool, which is usually used to determine what patients would benefit from CGA, was also assessed [22]. A cut-off less or equal to fourteen is usually admitted to identify vulnerable patients who need a CGA [22].

Cognition was evaluated using the Mini Mental State Evaluation (MMSE) [23]. A MMSE score of 24 or less was used to identify cognitive impairment [24]. The nutritional status was assessed by the Mini Nutritional Assessment (MNA) [25]. A MNA score ≥ 24 indicates a good nutritional status, a score of 17–23.5 indicates a risk of malnutrition and a score less than seventeen indicates malnutrition. The Hearing Handicap Inventory for the Elderly-Screening (HHIE-S), a self-assessment scale, was used to assess hearing loss [26]. An ophthalmologic evaluation was performed focusing on near vision (Parinaud chart), distance vision (Snellen chart) and detection of age-related macular degeneration (using Amsler grid). Visual impairment is defined using definitions detailed in a former work [27].

### Statistical analysis

We performed a descriptive analysis of the patients, cancers, and treatments characteristics. We performed a bivariate analysis to compare the CGA characteristics of the patients according to the change in cancer treatment plan (change or no change). Chi-square test or Fisher exact test were used for qualitative variables, and Student’s t test (in case of normal distribution) or the Mann-Whitney non-parametric test were used for quantitative variables. A multivariate logistic regression, using backward selection, was performed to test the association between CGA components and change in treatment decision. The multivariate model was built using variables which were associated in the bivariate analysis with a *p*-value< 0.20. Collinear variables were not entered in the final model. Interactions were tested according to clinical judgment. Statistical analyses were carried out using STATA version 11 (STATA Corp., TX USA).

## Results

A total of 452 patients, aged 65 or older, were referred to the GFC between October 2011 and January 2016 by their oncologist, hematologist, surgeon or radiation therapist. Thirty four patients were excluded from the analysis: six because their diagnosis of cancer was uncertain or still under investigation; seventeen due to poor health status of the patient where a complete clinical assessment was not feasible; and eleven had a diagnosis of hematologic disease without specific treatment plan (10 myelodysplastic syndrome and 1 monoclonal gammopathy), and were not evaluated specifically in the context of their hematologic diseases but for geriatric issues. Patients referred by their hematologist with myelodysplastic syndrome (MDS) and a systemic anti-MDS treatment plan were kept in the analysis. Descriptive statistics and demographic variables of the 418 remaining patients with cancer are described in Tables 1 and 2.

**Table 1:** Descriptive analysis of the 418 oncogeriatric patients evaluated at the Geriatric Frailty Clinic: socio-demographic characteristics and CGA domains

Characteristics	Mean ± SD or N (%)
**Socio-demographic characteristics**	
Age (years)	82.8 ± 5.5
Gender (female)	201 (48.1)
Living conditions (*n* = 412)	
Live alone	147 (35.7)
Marital status	
Married (vs widowed, single or divorced)	203 (48.6)
Education (*n* = 415)	
No education or Elementary School	232 (55.9)
Middle/ High School/ College	183 (44.1)
**Medical characteristics**	
Charlson Comorbidity Index	1.4 ± 1.6
Number of drugs	5.7 ± 3.2
Drugs ≥5	255 (61.0)
G-8 score ≤ 14 (*n* = 397)	334 (84.1)
**Cognitive assessment**	
MMSE ≤24 (*n* = 405)	149 (36.8)
**Nutritional assessment**	
MNA (*n* = 411)	
MNA ≥ 24	169 (41.1)
MNA (17–23.5)	201 (48.9)
MNA < 17	41 (10.0)
**Physical assessment**	
ADL ≤ 5	118 (28.2)
IADL ≤7	326 (78.0)
SPPB (*n* = 414)	
10–12 (high performance)	126 (30.4)
7–9 (medium performance)	139 (33.6)
≤ 6 (low performance)	149 (36.0)
Gait speed (< 1 m/s)	324 (77.5)
Grip strength (kg)	
Women	16.1 ± 5.4
Men	26.6 ± 7.5
Fried’s criteria	
Robust (0 criteria)	33 (7.9)
Pre-frail (1–2 criteria)	155 (37.1)
Frail (≥ 3 criteria)	230 (55.0)
**Sensory assessment**	
HHIES score (*n* = 389)	
No hearing handicap	224 (57.6)
Mild-moderate hearing handicap	143 (36.8)
Significant hearing handicap	22 (5.7)
Visual deficit	211 (57.3)

*SD* Standard Derivation, *G-8* Geriatric-8 Score, *MMSE* Mini Mental State Examination, *MNA* Mini-Nutritional Assessment, *ADL* Activities of Daily Living, *IADL* Instrumental Activities of Daily Living, *SPPB* Short Physical Performance Battery, *HHIES* Hearing Handicap Inventory fort the Elderly – Screening

**Table 2:** Type and stage of cancer in the 418 oncogeriatric patients evaluated at the Geriatric Frailty Clinic

Characteristics	N (%)
**Type of cancers (solid cancers)**	**339 (81.1)**
**Digestive cancer**	**124 (29.7)**
Colorectal	86 (69.3)
Stomach	10 (8.1)
Pancreas cancer	11 (8.9)
Other (esophageal, hepatic, duodenal, cholangiocarcinoma …)	17 (13.7)
**Urologic cancer**	**77 (18.4)**
Bladder	41 (53.2)
Kidney	26 (33.8)
Prostate	10 (13.0)
**Gynecologic cancer**	**70 (16.8)**
Breast	52 (74.3)
Ovarian	9 (12.9)
Other	9 (12.9)
**Head and neck Cancer**	**24 (5.7)**
**Lung cancer**	**18 (4.3)**
**Skin cancer**	**10 (2.4)**
**Melanoma**	**3 (0.30%)**
**Non-melanoma**	**7 (0.70%)**
**Other**	**16 (3.8)**
**Cancer stage**	
Local	155 (37.1)
Loco-regional	75 (17.9)
Metastasis	109 (26.1)
**Hematological cancer**	**79 (18.9)**
Lymphoma	23 (29.1)
Acute myelogenous leukemia	1 (1.3)
Myelodysplastic syndrome	8 (10.1)
Multiple myeloma	13 (16.5)
Chronic lymphocytic leukemia	34 (43.0)

The most prevalent cancers in men were digestive cancers (34.1%) especially colorectal cancers (64.9% of digestive cancers). In women the most frequent cancers were gynecologic cancers (34.3%) and mainly breast cancer (75.4% of gynecologic cancers).

Most of the patients were assessed before surgery or chemotherapy (77.8%) (Table 3). Targeted therapies were proposed only to 7 patients (1.7%). Thirty-four patients were admitted only for supportive care treatment, with no cancer treatment plan defined (cancer treatment was already achieved for 17 patients, and not decided for the others).

**Table 3:** Reason for assessment and decision after the comprehensive geriatric assessment

Initial Cancer treatment plan	N (%)
Surgery	173 (41.4)
Chemotherapy	148 (35.4)
Adjuvant chemotherapy	18 (4.3)
Radiotherapy	11 (2.6)
Chemotherapy and surgery	4 (1.0)
Chemotherapy and radiotherapy	6 (1.5)
Surgery and radiotherapy	2 (0.5)
Hormonal therapy	13 (3.1)
Hormonal therapy and radiotherapy	1 (0.2)
Targeted therapy	6 (1.5)
Hormonal therapy and targeted therapy	1 (0.2)
Chemoembolization	1 (0.2)
Supportive care treatment	34 (8.1)
**Change in the initial cancer treatment plan (except for patients admitted for “supportive care treatment”) (*n*** **= 384)**	
No change	320 (83.3)
Change (decrease in chemotherapy intensity)	8 (2.1)
Change from specific cancer treatment to supportive care or less aggressive treatment	56 (14.6)

Among the 384 patients evaluated with an initial cancer treatment plan, the treatment was changed in 16.7% (*n* = 64) of patients. There was no proposal to increase the intensity of the initial cancer treatment plan in this sample. A decrease of the intensity of the chemotherapy (dose adaptation of the chemotherapy or proposition of a single agent therapy instead of a combination therapy) was suggested in 8 patients (2.1%). Supportive care was recommended in 32 patients (57.1%), and a less aggressive treatment was suggested for the others (mainly radiotherapy instead of surgery or “surgery and chemotherapy” in 13 patients (23.2%), and hormonal therapy instead of chemotherapy or surgery in 5 patients (8.9)).

After discussion with the referring practitioner, hhe initial cancer treatment plan was maintained in 6 patients (9.7%), and the change in cancer treatment was carried out by the oncologist in 56 patients (90.3%). One patient was lost to follow-up.

As for the 63 patients with a normal G-8 score (16% of the population included), the treatment was changed only for three patients (4.8% of the patients with a normal G-8 score).

In the bivariate analysis, age, cognitive function, nutrition, activities of daily living, instrumental activities of daily living, physical performance (SPPB), gait speed, Fried criteria and visual impairment were significantly associated with change in the initial cancer treatment plan. In the multivariate analysis, a MMSE score less than 24, a malnutrition assessed with a MNA score less than seventeen, and low physical performance (SPPB≤7) were significantly associated with change in the initial cancer treatment plan (Table 4). An interaction between physical performance (assessed by SPPB) and nutrition (assessed with MNA) was tested but was not significant (data not shown).

**Table 4:** Bivariate and multivariate association between change in the initial cancer treatment plan and comprehensive geriatric assessment

Characteristics	Bivariate OR (95% CI)	p	Multivariate OR (95% CI)	p
Age (years)	1.06 (1.01–1.11)	0.014	–	
Gender (female)	1.27 (0.74–2.17)	0.386	–	
Living conditions (Live alone)	1.07 (0.60–1.90)	0.827	–	
Marital status			–	
Married (vs widowed, single or divorced)	0.85 (0.49–1.46)	0.553		
Education (*n* = 438)			–	
Middle/ High School/ College (vs no education or elementary school)	0.71 (0.41–1.24)	0.235		
Charlson Comorbidity Index	1.06 (0.99–1.26)	0.466	–	
Number of drug (≥ 5)	1.77 (0.98–3.19)	0.057	–	
MMSE (≤24)	3.53 (1.98–6.29)	< 0.001	2.15 (1.13–4.12)	0.020
MNA (*n* = 429)				
MNA ≥ 24	1		1	
MNA (17–23.5)	2.45 (1.22–4.93)	0.012	1.44 (0.62–3.34)	0.391
MNA < 17	11.08 (4.68–26.22)	< 0.001	3.33 (1.18–9.37)	0.023
ADL (≤5)	4.64 (2.65–8.12)	< 0.001	–	
IADL (≤7)	7.12 ((2.17–23.30)	0.001	–	
SPPB				
10–12 (high performance)	1		1	
7–9 (medium performance)	1.91 (0.69–5.27)	0.210	1.50 (0.45–4.98)	0.511
≤ 6 (low performance)	9.56 (3.90–23.40)	< 0.001	4.55 (1.43–14.46)	0.10
Gait speed (< 1 m/s)	3.22 (1.34–7.75)	0.009		
Fried’s criteria			–	
Robust (0 criteria)	1			
Pre-frail (1–2 criteria)	0.39 (0.09–1.65)	0.200		
Frail (≥ 3 criteria)	3.21 (0.94–11.01)	0.063		
Hearing impairment	0.89 (0.49–1.60)	0.694	–	
Visual impairment	2.01 (1.06–3.83)	0.031	–	

*OR* Odds Ratio, *MMSE* Mini Mental State Examination, *MNA* Mini-Nutritional Assessment, *ADL* Activities of Daily Living, *IADL* Instrumental Activities of Daily Living, *SPPB* Short Physical Performance Battery, *HHIES* Hearing Handicap Inventory fort the Elderly – Screening

## Discussion

The decision-making process in older patients with cancer is challenging. In our study population, the initial cancer treatment plan was deemed inappropriate for 16.7% of patients (*n* = 64). A low MMSE score, malnutrition, and low physical performance were independently associated with change in the initial cancer treatment plan.

In previous studies exploring the impact of geriatric evaluations on treatment decisions in older patients with cancer, the oncologic treatment was modified in 8 to 54% of all patients (with a median of 28%) [14]. In our study, the initial cancer treatment plan was changed in only 16.7% of patients. This difference may be difficult to compare with previous studies because of the populations heterogeneity, the various types of cancer, the various geriatric evaluations and different settings [14]. Nevertheless, oncogeriatric evaluation has been implemented in routine clinical practice for a few years in our clinical setting [15], and may have influenced and improved decision-making in this discipline. In addition, geriatric treatment recommendations were closely followed-up by the oncologist when the initial treatment plan was changed (in 91.1% of patients).

In cancer treatment, malnutrition is a substantial parameter to consider, because of its association with treatment toxicity and mortality [28]. In our study, malnutrition is significantly associated with changes in planned cancer treatment. Our results are consistent with past studies exploring CGA parameters associated with change in cancer treatment decision. In two studies, a low BMI under 21 kg/m2 was associated with a modification of the cancer treatment plan [29, 30], and according to Caillet et al. malnutrition evaluated by MNA, BMI, weight loss or low serum albumin was also associated with changes in cancer treatment (mainly a decrease in treatment intensity) [31]. In our analysis, malnutrition was defined only with the MNA score, which may have underestimated the prevalence of malnutrition in this population.

Another CGA parameter significantly linked with change in cancer treatment plan is a MMSE score under 24. Many factors can explain the fact that cognitive impairment may trigger a change in cancer treatment, preferentially from an aggressive treatment to a less aggressive option. First, past studies suggest that older patients with cognitive impairment are less compliant with treatment, which could affect the benefit of chemotherapy for example [32]. Secondly, discussion and understanding regarding treatment options may be more complex in this population: this may jeopardize the choice of the treatment. When decision-making capacity is deteriorated, patients tend to choose preferentially the less aggressive option [33]. Third there is evidence that chemotherapy can worsen cognitive functions [34]. Furthermore cognitive impairment is associated with cancer mortality or the probability of not completing chemotherapy [35–37]. But the impact of cognitive impairment or dementia on chemotherapy tolerance, hospitalizations or patient-reported outcome measures remains insufficiently investigated and unclear [38]. To our knowledge, only one study concluded that a low MMSE score (< 26) was associated with change in cancer treatment plan, specifically in lung cancer [39]. The impact of pre-existing cognitive-impairment on cancer-related outcomes needs to be clarified to improve cancer decisions and care in older adults.

In this study, low physical performance defined by a SPPB score less or equal to six, is associated with change in the initial cancer treatment plan. Physical performance tests reflect well frailty in oncogeriatric patients and are easy and rapid to use in clinical settings [11]. In previous studies exploring the effect of CGA on treatment decisions, physical performance were not systematically tested or were sometimes limited to the number of falls [29, 30, 40]. According to Farcet et al., the number of Fried’s criteria was significantly associated with a modification of the initial cancer treatment plan [41]. In our study, frailty according to the same definition, was not significantly associated with change in treatment plan. As most of the patients were frail or prefail (only 8% were robust), this information is probably not relevant to clinicians. They prefer to base their judgment mainly on the results of the SPPB which seems to offer a better discrimination of subjects with poor physical performance. This is the first time that the SPPB score is identified as a test that could be useful to modify treatment decision in oncogeriatric patients. Its use in routine clinical practice should be considered when evaluating oncogeriatric patients.

This study has some limitations. First, some parameters usually assessed in a geriatric evaluation (such as mood, BMI, or weight loss), were not systematically recorded in our dataset and were not exploited in this analysis. Then, the geriatric assessment was operated in only one hospital, by the same medical team, which may prevent reproducible research in other clinical settings. Moreover, oncologists referred patients to the frailty clinic without using an identified screening tool, but mainly according to their clinical judgment (if the patient seems frail or not). The results of the G-8 assessment show that they referred mainly patients (84%) that needed a CGA. But on the other hand, they may have selected only the frailest patients, and overestimate the robustness of the other patients, who would have been identified as vulnerable with an assessment tool. Indeed, past studies have shown that oncologist’s ability to identify frailty only according to their clinical judgment was low compared to CGA [42, 43]. This is a potential bias in this study. Finally, a wide spectrum of cancer was included in this work, at different stages, with a wide range of treatments, and with different level of possible complications. It is possible that the decision-making process is different according to the type and stage of cancer, and the type of treatment planned. Specific studies should be planned in the most prevalent cancers and according to the type of treatment to establish specific guidelines in older patient with cancer. This study has also several strengths: this is one of the few studies to evaluate the role of physical performance (SPPB or gait speed) in treatment decision in oncogeriatric patients. We used only international validated tools to assess domains of the CGA, as part of a multidisciplinary evaluation [5], and the median age was relatively high (83 years) (in a previous review evaluating the effect of geriatric evaluation on treatment decisions and outcomes, the median age ranged from 74 to 83 years) [14].

## Conclusion

In conclusion, this study shows that nutrition, physical performance and cognition are geriatric factors significantly associated with change in cancer treatment decisions in oncogeriatric patients. Prospective studies are needed to confirm their impact on treatment tolerance, cancer mortality, disability and patient-related outcomes, especially in highly-prevalent cancers.

## Data Availability

The dataset used and analyse during the current study are available from the corresponding author on reasonable request.

## Abbreviations

ADL: Activities of Daily Living
BMI: Body Mass Index
CGA: Comprehensive Geriatric Assessment
GFC: Geriatric Frailty Clinic
HHIE-S: Hearing Handicap Inventory for the Elderly-Screening
MMSE: Mini-Mental State Examination
MNA: Mini-Nutritional Assessment
SIOG: International Society of Geriatric Oncology
SPPB: Short Physical Performance Battery
TUG: Timed Up and Go Test
